# Cutaneous port-site recurrence secondary to tumour seeding following implantation of an intraperitoneal chemotherapy access port for gastric cancer

**DOI:** 10.1515/pp-2020-0102

**Published:** 2020-05-15

**Authors:** Koy Min Chue, Dexter Yak Seng Chan, Jimmy B.Y. So

**Affiliations:** Department of Surgery, National University Health System, Singapore, Singapore; Division of General Surgery (Upper Gastrointestinal Surgery), University Surgical Cluster, National University Hospital, Singapore Singapore

**Keywords:** gastric cancer, intraperitoneal chemotherapy, metastases, neoplasm seeding

## Abstract

Intraperitoneal chemotherapy has shown promising results for the treatment of peritoneal carcinomatosis in gastric cancer. However, the implantation of an intraperitoneal chemotherapy port may be associated with catheter-related complications. The authors describe a case of cutaneous port-site recurrence secondary to tumour seeding from an intraperitoneal chemotherapy access port.

Intraperitoneal chemotherapy while promising can be associated with catheter-related complications. Herein, the authors showcase a case of cutaneous port-site recurrence.

A 65-year-old Chinese male was diagnosed with a gastric cardia adenocarcinoma with limited peritoneal disease. He was commenced on systemic capecitabine and oxaliplatin, with intraperitoneal paclitaxel via an intraperitoneal chemotherapy port. Following clinical resolution of the peritoneal disease after chemotherapy, he underwent a conversion salvage gastrectomy with D2 lymphadenectomy. Final histology showed ypT4aN0 diffuse-type gastric adenocarcinoma. Postoperative recovery was uneventful, and he was re-commenced on chemotherapy.

Following 10 cycles of chemotherapy, a staging scan noted new deposits in the peritoneal lining. There were also two palpable masses on the anterior abdominal wall (Figure 1). Punch biopsy confirms metastatic adenocarcinoma. He eventually demised 12 months after diagnosis.

This case highlights a rare complication following intraperitoneal chemotherapy port insertion. Patients should be warned of possible port-site recurrence as a result of tumour dissemination.

**Figure 1:**
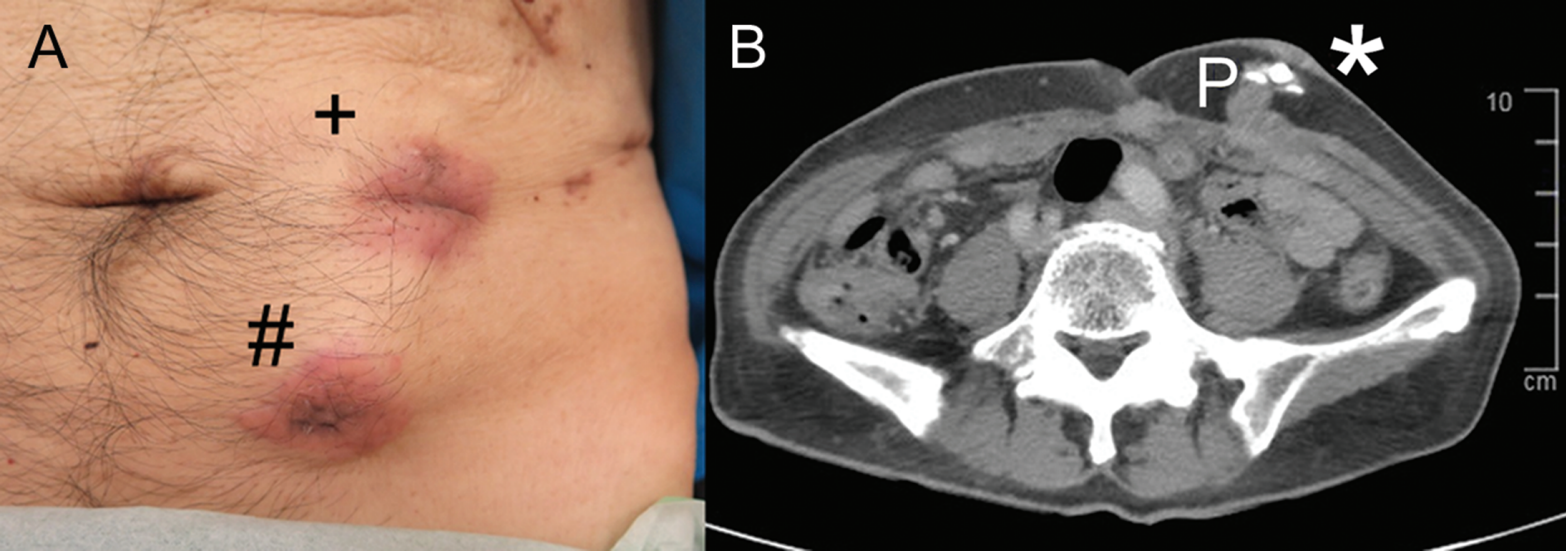
Cutaneous port-site recurrence. (A) Erythematous, indurated, hard masses on the skin from cutaneous port-site recurrence. Lesion marked “+” was the site of the subcutaneously placed intraperitoneal chemotherapy access port. Lesion marked “#” was the site of the 5 mm trocar for previous diagnostic laparoscopy.(B) Axial computer tomography scan images showing the cutaneous recurrences adjacent to the intraperitoneal chemotherapy access port.The asterisk (*) denotes the cutaneous recurrences while P denotes the intraperitoneal chemotherapy access port.

